# Dr. Beddoes's Queries

**Published:** 1801-07

**Authors:** Thomas Beddoes


### *To the Editors of the Medical and Physical Journal.*

Gentlemen,

In no inquisitive age has there existed a moment more favourable than the present for illustrating a great physiological problem; I mean the influence of the condition of parents on the number and condition of the progeny. One cannot help fearing that, in consequence of the long and severe scarcity, a degradation degradation of the ſpecies, partially begun in certain manufactories, will become general and permanent among us.

The political œconomiſt will attend to the proportion of births and marriages during this moſt diſaſtrous period. The medical philoſopher will look more cloſely into the ſubject. Nor will any thing ſhort of a body of information concerning the ſtate of new-born infants ſatisfy him. Without ſtopping therefore to demonſtrate the propriety of the inveſtigation, I propoſe to thoſe who have opportunities of obſervation the following queries:

1. Have the children of the poor of late come into the world more puny, meagre, or ſmaller, than in more plentiful times?

2. Have they appeared to die in greater numbers ſhortly after birth, and with what preceding ſymptoms?

I alſo wiſh to know,

3. Whether miſcarriages have been more frequent among this claſs? And,

4. Whether the mothers have recovered more ſlowly, and been ſubject to greater loſſes of blood?

*June* 7, 1801. THOMAS BEDDOES.

